# Association between Asthma and Suicidality in 9–12-Year-Old Youths

**DOI:** 10.3390/brainsci12121602

**Published:** 2022-11-23

**Authors:** Kevin W. Hoffman, Elina Visoki, Stirling T. Argabright, Laura M. Schultz, Grace E. Didomenico, Kate T. Tran, Joshua H. Gordon, Barbara H. Chaiyachati, Tyler M. Moore, Laura Almasy, Ran Barzilay

**Affiliations:** 1 Department of Psychiatry, Perelman School of Medicine, University of Pennsylvania, Philadelphia, PA 19104, USA; 2 Lifespan Brain Institute, Children’s Hospital of Philadelphia and Penn Medicine, Philadelphia, PA 19104, USA; 3 Department of Child and Adolescent Psychiatry and Behavioral Science, Children’s Hospital of Philadelphia, Philadelphia, PA 19104, USA; 4 Department of Biomedical and Health Informatics, Children’s Hospital of Philadelphia, Philadelphia, PA 19104, USA; 5 PolicyLab, Clinical Futures, Division of General Pediatrics, Children’s Hospital of Philadelphia, Philadelphia, PA 19104, USA; 6 Department of Genetics, Perelman School of Medicine, University of Pennsylvania, Philadelphia, PA 19104, USA

**Keywords:** suicidality, asthma, immune dysregulation, inflammation, adolescents, ABCD Study

## Abstract

Purpose: Suicidal ideation and attempts in youth are a growing health concern, and more data are needed regarding their biological underpinnings. Asthma is a common chronic inflammatory disorder in youth and has been associated with suicidal ideation and attempts in adolescent and adult populations, but data in younger children and early adolescents are lacking. We wished to study associations of asthma with childhood suicidality considering asthma’s potential as a clinically relevant model for childhood chronic immune dysregulation. Methods: Using data from the Adolescent Brain Cognitive Development (ABCD) Study (*n* = 11,876, 47.8% female, mean age 9.9 years at baseline assessment and 12.0 years at two-year follow-up), we assessed associations between asthma and suicidal ideation and attempts through baseline to two-year follow-up. Results: Asthma history as defined by parent report (*n* = 2282, 19.2% of study population) was associated with suicide attempts (SA) (odds ratio (OR) = 1.44, *p* = 0.01), and this association remained significant even when controlling for demographics, socioeconomic factors, and environmental factors (OR = 1.46, *p* = 0.028). History of asthma attacks was associated with both suicidal ideation (SI) and SA when controlling for demographics, socioeconomic factors, and environmental factors (OR = 1.27, *p* = 0.042; OR = 1.83, *p* = 0.004, respectively). The association of asthma attack with SA remained significant when controlling for self-reported psychopathology (OR = 1.92, *p* = 0.004). The total number of asthma attacks was associated with both SI and SA (OR = 1.03, *p* = 0.043; OR = 1.06, *p* = 0.05, respectively). Conclusions: Findings suggest an association between asthma and suicidality in early adolescence. Further research is needed to investigate mechanisms underlying this relationship.

## 1. Introduction

Compromised physical health and inflammatory dysregulation are associated with suicidal behaviors [1,2,3]. Youth with asthma commonly possess both of these traits. Asthma, a chronic inflammatory illness marked by spasms of the bronchi in the lungs, has been independently associated with suicide risk in older adolescent and adult populations [4], but more data are needed in young children and early adolescents. Considering that asthma is the most common chronic disease in youth, with steadily increasing rates, understanding its relation to suicide risk in this population is critical [5]. Compounding this urgency is a steadily increasing rate of suicidal ideation and suicide attempts in preadolescents and youth of early adolescent age [6,7,8,9]. Indeed, investigation of suicidality and understanding its contributing factors in this age range is of paramount importance and requires large representative samples and careful methods [10]. 

Suicide attempts are correlated with the activation of immune-inflammatory and nitro-oxidative pathways that lead to inflammation and increased neurotoxicity and inversely correlated with several antioxidants and other neuroprotective factors [11]. This suggests a role for immune-driven neurotoxicity in the pathophysiology of suicide attempts. More broadly, immunologic changes including inflammatory dysregulation are associated with many psychiatric disorders, and several mechanisms have been proposed for this interaction, including inflammatory-mediated changes in neurotransmitter levels, brain cellular architecture, and cortisol signaling [12,13,14,15,16]. Given these observations, it is important to investigate psychiatric correlates in chronic inflammatory illnesses, including asthma, as possible disease sequela. 

A major challenge when studying the associations between asthma and suicidality is the need to account for many potential confounders. These confounders include demographics, socioeconomic status (SES), neighborhood and environmental factors, as well as psychiatric co-morbidities including anxiety and depression, all associated with both asthma and suicidality in youth [17,18,19]. Previous studies linking asthma and suicide in youth have relied on wide age ranges to be sufficiently powered for this analysis, which favors outcomes in older adolescents where suicidality is more common [20,21,22,23]. Indeed, identifying the prevalence of suicidality in children and young adolescents with asthma and the contributions of possible confounders is important for delivering better clinical care for this population and may provide insight into the wider role that chronic inflammatory illness plays in suicidality in youth. 

The purpose of this study was to evaluate the association of asthma with suicidality in youth aged 9–12 years. We used data obtained from the Adolescent Brain Cognitive Development (ABCD) Study [24], a large, diverse, genotyped youth sample (*n* > 11,000) with extensive phenotyping for sociodemographic factors, psychopathology, medical conditions and treatments, and multiple environmental exposures [25]. By using this large and extensively phenotyped dataset, we were able to address suicidality with high granularity, investigating suicidal ideation (SI) and suicide attempts (SA) as separate outcomes. We hypothesized that asthma, as an example of an immune dysregulation illness, would be associated with suicidality in this age of pre- and early adolescence. 

## 2. Methods and Materials

### 2.1. Study Design

We studied the association between asthma and suicidality using all available data from the ABCD Study that included three assessment waves from age 9 to 12. To address the inconsistency of suicidality reporting across time points (Supplementary Table S1) [26], we created “ever” variables for both the exposure (i.e., asthma measures) and suicidality measures (dependent variables), which is in line with prior work in this population [27].

#### 2.1.1. Sample

The sample is composed of all ABCD Study participants who completed the baseline assessment (*n* = 11,876). The ABCD Study sample includes a cohort of children aged 9–10 years at baseline, recruited through school systems [28]. Participants were enrolled at 21 sites, with a catchment area encompassing over 20% of the entire US population in this age group. We included data from the ABCD Study data release 4.0 (https://abcdstudy.org/, accessed on 22 August 2022), which includes data collected from baseline, 1-year, and 2-year follow-up. All participants gave assent. Parents/caregivers signed informed consent. The ABCD Study protocol was approved by the University of California, San Diego Institutional Review Board (IRB), and was exempted from a full review by the University of Pennsylvania IRB.

#### 2.1.2. Exposures

A history of asthma was based on parent report of an asthma diagnosis (variable medhx_2a from the ABCD Summary Scores Medical History) in any of the three ABCD Study assessments (baseline, year-1, and year-2 follow-up). We refer to this exposure as *asthma history* throughout the manuscript.

A history of asthma attack was based on parent report that the participant was seen by a healthcare provider for an asthma attack (variable medhx_6l from the ABCD Summary Scores Medical History) in any of the three ABCD Study assessments (baseline, year-1, and year-2 follow-up). We refer to this exposure as *asthma attack* throughout the manuscript.

Asthma severity was estimated by the total number of parent-reported clinical visits due to asthma attacks (medhx_ss_6l_times_p, continuous variable, assessed as asthma attacks since last follow-up in year-1 and year-2 follow-up) compiled across all assessed timepoints.

#### 2.1.3. Outcome Measures

The ABCD Study assessed suicidal ideation and attempt (past or current) as part of the validated and computerized Kiddie-Structured Assessment for Affective Disorders and Schizophrenia for DSM-5 (KSADS-5) [29]. Items relating to self-injurious behavior without suicidal intent were not included in the current analysis. We separated suicidality into two measured outcomes: suicidal ideation (SI) and suicide attempt (SA). Prevalence of SI and SA is detailed in Table 1. As prior studies (including in the ABCD Study) showed poor youth–caregiver agreement on suicidal ideation and attempts [30,31,32], we focused on child report of SI and SA in the current analysis.

#### 2.1.4. Covariates

Covariates were obtained from ABCD Study baseline data and included demographics: age, sex, race (Black, White, Other), and Hispanic ethnicity (used in Model 1, described below); household-level socioeconomic status (SES) variables: household income, average parent education, and maternal age; neighborhood-level factors: area deprivation index, population density, NO_2_ and PM 2.5 (annual average at 10 × 10 km^2^), and proximity to major roads (added in Model 2, described below). We used the youth-reported Brief Problem Monitor (BPM) (added in Model 3, described below) as a measure of general psychopathology.

#### 2.1.5. Other Measures

To evaluate the internal validity of the asthma variables, we tested their association with two asthma-related variables: (i) polygenic risk score of asthma (PRS-asthma); (ii) parent report of asthma medication use.

#### 2.1.6. PRS-Asthma

PRS-asthma was calculated using computational pipelines previously described by our group [33] using the summary statistics of a recent GWAS of asthma in a multi-ancestry population [34]. Briefly, PRS-CS (PRS using SNP effect sizes under continuous shrinkage) [35] was used to infer posterior effect sizes of the SNPs in the dataset that overlapped with the asthma GWAS summary statistics and an external 1000 Genomes linkage disequilibrium (LD) panel matched to the ancestry group used for the GWAS. PRSs for the European ancestry (EUR) and African ancestry (AFR) subsets of the ABCD Study^®^ participants were computed using the corresponding ancestry LD panel and GWAS. Raw PRSs were produced by PLINK 1.9 and then standardized and ancestry-corrected in R.

#### 2.1.7. Medication Use

Asthma medications taken in the past 2 weeks (binary variable) as reported at baseline ABCD Study assessment included: beta-agonists, inhaled corticosteroids, leukotriene modifiers, and others (see Supplementary Table S2 for a full list of asthma medications). We include systemic steroids (nontopical corticosteroids) and antidepressants including SSRI, SNRI, and other atypical antidepressants as additional covariates in Supplementary Tables S3 and S4.

### 2.2. Statistical Analysis

We used R for our data analyses. Mean (standard deviation (SD)) and frequency (%) were reported for descriptive purposes. Univariate comparisons were made using *t*-tests or chi-square tests, as appropriate. We corrected for multiple comparisons using a Bonferroni method to adjust *p*-values and confidence intervals when testing associations of exposures with suicidal ideation and suicide attempts. We employed listwise deletion for participants with missing data, the rate of which was lower than 2.2% for all variables except neighborhood-level variables (5.5–7.4%), household income (19.8%), parents’ education (5.7%), suicide attempt (15.9%), suicide ideation (14.0%), and asthma PRS (36.4%).

The analytic plan and hypotheses were preregistered on Open Science Framework (https://osf.io/nxeys/, accessed on 20 November 2022) in July 2021, and analyses were conducted between September 2021 and August 2022, following the release of the ABCD Study data drop 4.0. Data preprocessing and analysis are detailed at https://github.com/barzilab1/ABCD_asthma_inflammation_k (accessed on 20 November 2022).

### 2.3. Validation of the Asthma Phenotype in ABCD Study

We employed two methods to internally validate the asthma measures that we used: First, we tested the association of PRS-asthma with the asthma phenotype. Second, we tested the association of the asthma phenotype with an indicator that the participant was taking asthma medications.

### 2.4. Main Analyses

We tested a set of mixed-effects logistic regression models with asthma exposures as independent variables and suicidality (SI or SA) as the dependent variable. These models took the hierarchical nature of the ABCD data into account by including a 2-level hierarchy where subject IDs were first nested according to the family ID and then nested according to the research site ID. Moreover, we assumed a similar time trend across all subjects and only allowed random intercepts in all mixed models. This approach allowed us to account for the random variability across families and study sites. More specifically, the random intercepts allow the outcome (SI/SA rates) to be higher or lower for each family and each site, allowing us to account for site variation. We first tested models using the baseline, year-1, and year-2 follow-up data with asthma, using asthma history as a “broad phenotype” exposure and asthma attack as a more specific asthma exposure.

In all the above-described models, we added covariates in a stepwise manner, beginning with demographics (Model 1), then adding household-level socioeconomic factors and neighborhood-level exposures (Model 2). To account for general psychopathology, the total score of the youth-reported BPM was added to Model 2 (Model 3).

To address the possibility that severity of asthma affects the associations identified in the main analyses, we tested models using a proxy of asthma severity (“number of asthma attacks”) as the independent variable.

Finally, we further co-varied for systemic steroids and antidepressants as a supplementary, exploratory analysis to consider medications used by this population that may influence suicidality.

#### Sensitivity Analysis

To address specificity of the relationship between the study’s exposure (asthma) and suicidality, we ran similar models as described above, only this time we used the measure “ever seen a doctor for broken bones” (measure medhx_6a in ABCD Study) as the independent variable, instead of asthma.

To address potential confounding effects of medications, we reran the main models co-varying for treatment with systemic steroids or with antidepressants.

## 3. Results

### 3.1. Prevalence of Asthma and Suicidality in ABCD

A total of 11,876 youth were assessed over the first three waves of the ABCD Study assessment (mean age = 12.0 years at two-year follow-up (SD = 0.66), 5681 female (47.8%)). Of these, 2282 (19.2%) endorsed a history of asthma (assessed by caregiver report), while 816 (6.9%) had been seen by a medical provider for an asthma attack. A total of 2022 (17.0%) youth reported history of SI, and 354 (3.0%) reported a history of SA. Demographic and clinical characteristics of the baseline sample are detailed in Table 1.

### 3.2. Validation of the Asthma Phenotype in ABCD Study

PRS-asthma was positively associated with asthma history (odds ratio (OR) = 1.33, 95% confidence interval (CI) = 1.24–1.43, *p* < 0.001 for the meta-analyzed model comprising African and European ancestry participants, accounting for age, sex, race, and ethnicity and for 10 genetic principal components). Similarly, history of asthma attack was also associated with a greater asthma PRS (OR = 1.48, CI = 1.33–1.64, *p* < 0.001). Full models’ statistics are detailed in Table 2.

Asthma history was also positively associated with use of asthma medications (OR = 11.39, CI = 5.85–22.18, *p* < 0.001, model accounts for age, sex, races (Black, White, and others), and ethnicity).

### 3.3. Association of Asthma History with Suicidality

Participants with asthma history reported more SI and more SA than their peers did (Table 1, 19.0% vs. 16.6%, *p* = 0.007 and 3.9% vs. 2.8%, *p* = 0.01, respectively). In contrast, there was no difference in rates of SI and SA between participants who had ever broken bones (*n* = 2256, of which 18.3% endorsed SI and 3.1% endorsed SA) and those who had not (*n* = 9617, of those 20.2% endorsed SI and 3.7% endorsed SA, *p* = 0.518 and *p* = 0.150, comparing SI and SA, respectively).

**Figure 1:**
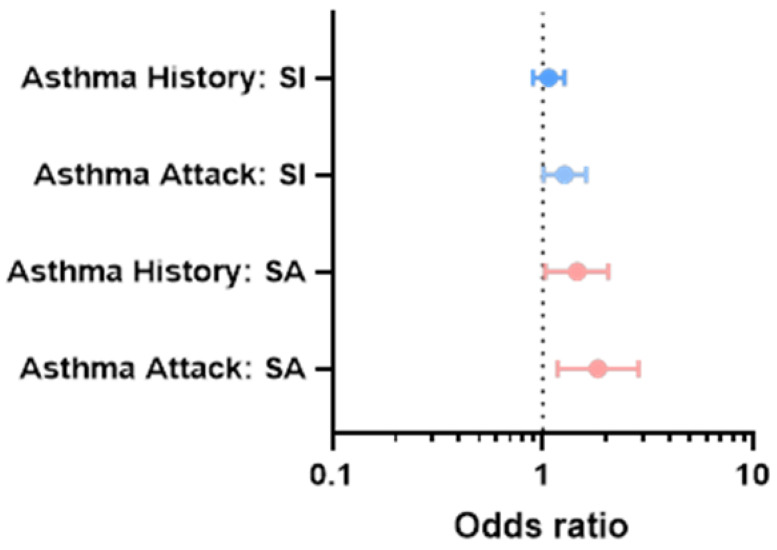
Effect sizes of the associations between parent report of asthma, asthma attack, suicidal ideation, and suicide attempt. Caption: bars represent the odds ratio and 95% confidence intervals of models co-varying for demographics, household-level socioeconomic factors, and neighborhood-level factors. The odds ratio increases for more severe presentations of asthma (attack vs. history) and for more severe presentations of suicidality (SA vs. SI). Abbreviations: SI = suicidal ideation; SA = suicide attempt.

To address the specificity of the association between asthma and suicidality, we included covariates to account for potential confounding effects (Figure 1, Table 3 and Table 4). Asthma history was not associated with SI in any of the multivariate models (all *p*’s ≥ 0.6, Table 3). Asthma history was significantly associated with SA even when co-varying for demographic factors, household SES, as well as neighborhood and environment confounders (Table 4, Model 2: OR = 1.46, CI = 1.03–2.05, *p* = 0.028), but it was no longer significant when co-varying for self-reported overall psychopathology (Table 4, Model 3: OR = 1.33, CI = 0.92–1.93, *p* = 0.162). Additionally, co-varying for systemic steroids or antidepressants did not significantly alter the results (Supplementary Tables S3 and S4).

### 3.4. Association of Asthma Attacks with Suicidality

Asthma attack was significantly associated with SI when co-varying for confounders including demographic factors, household SES, and neighborhood and environment confounders (Figure 1, Table 3, Model 2: OR = 1.27, CI = 1.01–1.61, *p* = 0.042), but was not significant when further co-varying for self-reported overall psychopathology (Model 3: OR = 1.28, CI = 0.99–1.66, *p* = 0.066). Asthma attack was significantly associated with SA for all covariates including self-reported overall psychopathology (Table 4, Model 3: OR = 1.92, CI = 1.20–3.09, *p* = 0.004). Additionally co-varying for systemic steroids or antidepressants did not significantly alter the results (Supplementary Tables S3 and S4).

To evaluate a dose response, we tested whether the total number of asthma attacks was associated with SA and SI. For patients who reported at least one lifetime asthma attack, the odds for endorsing SI and SA were more likely for each additional asthma attack, OR = 1.03, *p* = 0.043; OR = 1.06, *p* = 0.05, respectively (model co-varied for age, sex, races, and ethnicity). 

## 4. Discussion

We describe an association between history of asthma and suicidality in a large, diverse sample of pre- and early adolescent US youth. This association was significant when accounting for multiple socioeconomic household variables and neighborhood confounders. The association of asthma attack with suicide attempt further remained significant when accounting for overall psychopathology. Notably, sensitivity analyses showed a lack of association between suicidality of a noninflammatory medical condition (bone fractures), further supporting the specificity of our findings. These results add to existing literature on the associations between asthma and suicidality in older adolescents [22,23,36] and adults [37,38,39,40,41], and support the biological evidence for an association between immune dysregulation and suicidality [2,42], presenting early in the lifespan. Taken as a whole, our findings provide a clinical indication for the link between inflammation (i.e., asthma) and suicidality at a developmental age when suicidality emerges. 

We found that asthma attacks were associated with suicide attempts even when co-varying for self-reported overall (nonsuicidality) psychopathology. This finding may suggest specificity in the relationship between asthma and suicide attempt risk that is over and above nonspecific psychopathology. This finding may have two important implications. First, from a clinical perspective, it suggests that for at least the youth described by this study, a commonly practiced strategy of screening for suicidality in individuals who endorse symptoms of depression may not be sufficient to capture those at risk of suicide attempt, particularly for those youth with a history of asthma attacks requiring medical attention [43]. This result highlights the importance of further investigation into this connection to both better understand suicidality in youth and develop better screening tools. Second, from a mechanistic research perspective, our study suggests that asthma can be used as a clinical test case for studying the link between chronic inflammatory conditions and youth suicide risk. Future works can use this conceptual model to better understand the ties between early life suicidality and stress exposure [44,45], which in turn is associated with immune dysregulation [46].

A key aspect of this study is the definition of asthma phenotype using observational data from a study that was not focused on asthma and did not include a clinical asthma evaluation. Previous research has shown that parent report of asthma, while less stringent than clinical evaluation, is a reliable estimate of asthma prevalence [47]. Our analyses of internal validation of the asthma phenotype with genetic data and with medication use support the notion that parent report is valid. Our use of genetic risk is particularly notable because the polygenic risk was developed in a separate adult population, but still shows significant association with the parent report asthma phenotype in the ABCD Study. Indeed, we suggest that the asthma phenotype we used here is robust.

While the ABCD Study was not created with precise measures of asthma severity in mind, participants were asked about the total number of asthma attacks requiring medical attention, which provides a crude measure of asthma severity. Though imperfect, the significant correlation between number of asthma attacks and both suicidal ideation and suicide attempts suggests that more severe asthma increases risk of suicidality. Additionally, we examined and compared two different measures of asthma: asthma history and history of asthma attack. The asthma history phenotype captured nearly three times as many youth as those determined to have a history of asthma attack, likely due to a combination of both including youth with weaker asthma phenotypes (i.e., those diagnosed with asthma who never had an asthma attack requiring medical evaluation) as well as those youth with a history of asthma-like symptoms who would nonetheless fail to meet diagnostic criteria for asthma. Therefore, it is likely that the subgroup of youth reporting asthma attack is both more specific for a clinical threshold asthma phenotype as well as representative of a more severe asthma presentation.

The ABCD Study provides a valuable opportunity to study the relationship between medical conditions and mental health. A major strength is that its large sample size and deep phenotyping allow sufficient power to account for many potential confounders and support the specificity of our findings showing a positive association of asthma and childhood suicidality. While asthma is common, it is not evenly distributed across the population, with a different prevalence between genders, racial groups, socioeconomic backgrounds, and populations from urban vs. rural settings [48]. Similarly, gender, race, and socioeconomic factors are all associated with various levels of discrimination, which are all associated with youth suicidality [49,50,51]. Thanks to the study’s size and design, we could account for these confounders and still make a significant observation linking a medical condition to suicidality. While useful for asthma, this further suggests an ongoing and largely untapped role for the ABCD Study to analyze the often-complex relationships between many chronic medical illnesses and mental health.

This work and previous works in older populations establish an association between asthma and suicidality, but more research is needed to better understand the mechanisms underlying this association. A few potential pathways can explain this association. For example, it is possible that asthma symptoms are themselves distressing for many individuals and may therefore lead to an increased tendency for suicidal thoughts or behaviors, via reduced quality of life or increased distress, but it is also possible that other factors related to asthma, including the associated immune dysregulation, are important for suicidality. These different explanations can be framed as whether the “state” (i.e., active asthma symptoms) or “trait” of asthma (i.e., the history of asthma diagnosis) is causing the observed asthma–suicidality association. One way to address this question would be to compare suicidality for individuals with the state of having asthma to the proximity of an asthma attack versus other measures, including measures of inflammation. While the ABCD Study currently lacks the granularity to carry out such a study, future works can address this using more granular data on asthma severity and immune markers. Finally, it is important to consider that asthma medications may themselves influence suicidality. While in our present study we considered asthma medications as a method for validating asthma diagnosis in the ABCD Study population, it will be important to consider the effect of medications in future studies. In our sensitivity analysis considering systemic steroids, we did not see significant changes in outcomes when co-varying for steroids. It is important to note that while the ABCD Study did not collect data on medication dosage or duration of use, generally systemic steroid dosing tends to be lower for asthma than for other uses.

### Limitations

Several limitations should be considered when viewing our findings. First, asthma and its severity were established without a clinician’s rating and/or specific asthma scales, and we relied instead on parent report of asthma diagnosis, and report of asthma attacks. We suggest that the association of the phenotype with asthma PRS and with asthma medication use supports the validity of the asthma phenotype. Second, the evaluation of suicidality in young children is challenging, as evident by the inconsistency in endorsing suicidality across the three assessment waves. We made use of the KSADS instrument to assess SI and SA, which while highly studied, validated, and used for suicide research, was not specifically designed to assess suicidality. Third, while we co-varied for a dimensional measure of self-reported psychiatric distress, we did not include psychiatric diagnoses in our models, as these were not available for all study timepoints. Future longitudinal analyses of ABCD Study data could investigate the relationship between asthma, psychiatric disorders, and suicidal ideation and attempts. Lastly, while the ABCD Study is large, our findings need to be generalized and replicated in other youth datasets to establish external validity.

## 5. Conclusions

To conclude, this study describes an association between history of asthma and suicidality in a large community sample of preadolescent through early adolescent youth. The large sample, the within-sample diversity, and the broad nature of data collected allowed controlling for multiple confounders, providing a basis to investigate mechanisms underpinning this asthma–suicidality association. Our findings are in line with previous studies in older populations of adolescents and adults, demonstrating that asthma associates with suicidality even at early ages. Further, this association exists even when common confounders, including psychopathology, gender, socioeconomic factors, and neighborhood-level factors are considered. Findings highlight the importance of suicidality screening in youth with asthma and underscore the importance of ongoing work to investigate the biological and psycho-social underpinnings that may influence suicidality in this population.

## Figures and Tables

**Table 1:** Sample statistics for sociodemographics, asthma, and suicidality prevalence in the ABCD study.

	Total Population ^a^ *n* = 11,876	No Asthma *n* = 9591	Asthma *n* = 2282	*p*-Values
Age at the latest time point (2-year follow-up), mean (SD)	12.00 (0.66)	12.00 (0.66)	12.01 (0.67)	0.5500
Sex (female), *n* (%)	5681 (47.8)	4765 (49.7)	914 (40.1)	<0.00001
Black race, *n* (%)	2518 (21.2)	1773 (18.5)	743 (32.6)	<0.00001
White race, *n* (%)	8804 (74.1)	7300 (76.1)	1503 (65.9)	<0.00001
Asian race, *n* (%)	751 (6.3)	626 (6.5)	125 (5.5)	0.0714
Hispanic ethnicity, *n* (%)	2411 (20.3)	1940 (20.2)	471 (20.6)	0.6129
Household income, mean on an ordinal scale from 1–10 (SD)	7.54 (2.30)	7.65 (2.25)	7.07 (2.47)	<0.00001
Parent education, mean years (SD)	16.50 (2.63)	16.59 (2.64)	16.15 (2.57)	<0.00001
Maternal age at participants’ birth, mean (SD)	29.40 (6.27)	29.58 (6.21)	28.62 (6.49)	<0.00001
Area Deprivation Index: national percentiles, mean (SD)	40.04 (26.97)	38.94 (26.62)	44.64 (27.96)	<0.00001
Adjusted population density, mean (SD)	2212.95 (2777.16)	2195.18 (2759.64)	2290.04 (2850.74)	0.1547
3 years average of ground level NO_2_ at 10 × 10 km^2^, mean (SD)	2.45 (1.67)	2.47 (1.68)	2.39 (1.61)	0.071
Annual average of PM 2.5 at 10 × 10 km^2^, mean (SD)	7.53 (2.57)	7.50 (2.56)	7.67 (2.60)	0.00588
Proximity to major roads, in meters, mean (SD)	1185.68 (1281.58)	1184.09 (1299.05)	1192.03 (1205.15)	0.7963
Asthma attack, *n* (%)	927 (7.8)	33 (0.3)	894 (39.2)	<0.00001
Suicide ideation, *n* (%)	2022 (17.0)	1589 (16.6)	433 (19.0)	0.00465
Suicide attempt, *n* (%)	354 (3.0)	265 (2.8)	89 (3.9)	0.00785
Mean scores of average BPM scores across assessments, mean (SD)	7.02 (5.25)	6.92 (5.22)	7.42 (5.35)	0.00012

^a^ Total population at baseline.

**Table 2:** Association of PRS-asthma ^a^ with asthma phenotypes used in this study.

	African Ancestry (*n* = 1567)			European Ancestry (*n* = 5642)			Meta Analyzed Sample (*n* = 7209)		
	*Odds Ratios*	*95% CI*	*p*	*Odds Ratios*	*95% CI*	*p*	*Odds Ratios*	*95% CI*	*p*
Asthma History	1.08	0.96–1.21	0.222	1.49	1.37–1.62	**<0.001**	1.332	1.2447–1.4253	**<0.0001**
Asthma Attack	1.12	0.96–1.31	0.153	1.81	1.59–2.06	**<0.001**	1.4835	1.3430–1.6386	**<0.0001**

^a^ PRS-asthma derived from an asthma PRS originally developed in a large, racially diverse population [34].

**Table 3:** Association between asthma and suicidal ideation.

	Model 1 ^a^			Model 2 ^b^			Model 3 ^c^		
	Odds Ratio	95% CI	*p*	Odds Ratio	95% CI	*p*	Odds Ratio	95% CI	*p*
Asthma history	**1.07**	**0.92–1.25**	**0.610**	**1.07**	**0.90–1.27**	**0.732**	**1.04**	**0.86–1.25**	**1**
Asthma attack	**1.24**	**1.00–1.53**	**0.056**	**1.27**	**1.01–1.61**	**0.042**	**1.28**	**0.99–1.66**	**0.066**

^a^ Model 1 co-varies for demographics: age, sex, race (Black, White, Other) and Hispanic ethnicity. ^b^ Model 2 co-varies for demographics, household-level socioeconomic factors (income, average parent education, and maternal age), and neighborhood-level factors (area deprivation index, population density, NO_2_, PM 2.5, and proximity to major roads). ^c^ Model 3 includes all covariates from Model 2 and further co-varies for general self-reported psychopathology (BPM score). Abbreviations: OR = odds ratio; CI = confidence interval.

**Table 4:** Association between asthma and suicide attempt.

	Model 1 ^a^			Model 2 ^b^			Model 3 ^c^		
	Odds Ratio	95% CI	*p*	Odds Ratio	95% CI	*p*	Odds Ratio	95% CI	*p*
Asthma history	**1.37**	**1.00–1.87**	**0.046**	**1.46**	**1.03–2.05**	**0.028**	**1.33**	**0.92–1.93**	**0.162**
Asthma attack	**1.59**	**1.05–2.40**	**0.024**	**1.83**	**1.18–2.86**	**0.004**	**1.92**	**1.20–3.09**	**0.004**

^a^ Model 1 co-varies for demographics: age, sex, race (Black, White, Other) and Hispanic ethnicity. ^b^ Model 2 co-varies for demographics, household-level socioeconomic factors (income, average parent education, and maternal age), and neighborhood-level factors (area deprivation index, population density, NO_2_, PM 2.5, and proximity to major roads). ^c^ Model 3 includes all covariates from Model 2 and further co-varies for general self-reported psychopathology (BPM score). Abbreviations: OR = odds ratio; CI = confidence interval.

## Data Availability

Data used in the preparation of this article were obtained from the Adolescent Brain Cognitive Development (ABCD) Study (https://abcdstudy.org, accessed on 20 November 2022), held in the NIMH Data Archive (NDA).

## Supplementary Materials

The following supporting information can be downloaded at: https://www.mdpi.com/article/10.3390/brainsci12121602/s1, Table S1: Mismatch in suicidality and asthma reports across study assessments; Table S2: Asthma medications list; Table S3: Association between asthma and suicidal ideation co-varying for medications; Table S4: Association between asthma and suicide attempt co-varying for medications.

## Abbreviations

ABCD Study	Adolescent Brain Cognitive Development Study
IRB	Institutional Review Board
SI	suicidal ideation
SA	suicide attempt
BPM	Brief Problem Monitor
