# Supplementary material for: Association between Asthma and Suicidality in 9–12-Year-Old Youths
Source: Brain Sci. 2022 Nov 23;12(12):1602. doi: 10.3390/brainsci12121602 (PMC9775696; doi:10.3390/brainsci12121602)
Supplement: Supplementary file 1 [file brainsci-12-01602-s001.zip › brainsci-1977401-supplementary.pdf]

Supplementary Material: Association between asthma and suicidality in 9-12-year-old youths

Supplementary Table S1. Percent mismatch of suicidal ideation (SI) and suicide attempt (SA) and asthma (history of asthma and asthma attack) across time points (baseline, 1-year, and 2-year follow-up assessments).

	**% of mismatch**
**SI**	**17.82%**
**SA**	**3.19%**
**Asthma history**	**23.54%**
**Asthma attack**	**11.98%**

Supplementary Table S2. Asthma medications as reported by caregivers in the ABCD Study baseline assessment.

**Asthma Medications**
Albuterol
Levalbuterol
Formoterol
Salmeterol
Montelukast
Fluticasone propionate
Flunisolide
Budesonide
Beclometasone
Ciclesonide
Tiotropium bromide
Mometasone furoate
Triamcinolone Inhalant

Supplementary Table S3. Association between asthma and suicidal ideation co-varying for medications.

(A) All models include use of systemic steroids as a covariate.

	Model 1[a]			Model 2[b]			Model 3[c]		
	**OR**	**95%CI**	**p**	**OR**	**95%CI**	**p**	**OR**	**95%CI**	**p**
Asthma history	1.08	0.93-1.26	0.530	1.07	0.91-1.27	0.680	1.04	0.87-1.26	1
Asthma attack	1.25	1-1.55	0.044	1.28	1.01-1.62	0.038	1.29	0.99-1.67	0.056

(B) All models include use of antidepressant as a covariate.

	Model 1[a]			Model 2[b]			Model 3[c]		
	**OR**	**95%CI**	**p**	**OR**	**95%CI**	**p**	**OR**	**95%CI**	**p**
Asthma history	1.06	0.91-1.23	0.854	1.06	0.89-1.25	0.954	1.04	0.86-1.25	1
Asthma attack	1.22	0.98-1.51	0.084	1.26	0.99-1.6	0.056	1.27	0.98-1.65	0.076

[a]Model 1 co-varies for demographics: age, sex, race (Black, White, Other) and Hispanic ethnicity. [b]Model 2 co-varies for demographics, household-level socioeconomic factors (income, average parent education, and maternal age), and neighborhood-level factors (area deprivation index, population density, NO2, PM 2.5, and proximity to major roads). [c]Model 3 includes all covariates from Model 2 and further co-varies for general self-reported psychopathology (BPM score). Abbreviations: OR = odds ratio; CI= confidence interval.

Supplementary Table S4. Association between asthma and suicide attempt co-varying for medications.

(A) All models include use of systemic steroids as a covariate.

	Model 1[a]			Model 2[b]			Model 3[c]		
	**OR**	**95%CI**	**p**	**OR**	**95%CI**	**p**	**OR**	**95%CI**	**p**
Asthma history	1.39	1.02-1.9	0.034	1.48	1.05-2.08	0.022	1.35	0.93-1.95	0.142
Asthma attack	1.62	1.07-2.45	0.018	1.87	1.2-2.92	0.004	1.97	1.22-3.16	0.002

(B) All models include use of antidepressant as a covariate.

	Model 1[a]			Model 2[b]			Model 3[c]		
	**OR**	**95%CI**	**p**	**OR**	**95%CI**	**p**	**OR**	**95%CI**	**p**
Asthma history	1.32	0.96-1.81	0.106	1.40	0.98-1.99	0.062	1.32	0.91-1.93	0.184
Asthma attack	1.51	0.99-2.31	0.056	1.79	1.13-2.81	0.008	1.88	1.16-3.05	0.006

[a]Model 1 co-varies for demographics: age, sex, race (Black, White, Other) and Hispanic ethnicity. [b]Model 2 co-varies for demographics, household-level socioeconomic factors (income, average parent education, and maternal age), and neighborhood-level factors (area deprivation index, population density, NO2, PM 2.5, and proximity to major roads). [c]Model 3 includes all covariates from Model 2 and further co-varies for general self-reported psychopathology (BPM score). Abbreviations: OR = odds ratio; CI= confidence interval.
